# Further Evidence of Autosomal Recessive Inheritance of *RPL3L* Pathogenic Variants with Rapidly Progressive Neonatal Dilated Cardiomyopathy

**DOI:** 10.3390/jcdd9030065

**Published:** 2022-02-22

**Authors:** Hemanth Nannapaneni, Stephanie Ghaleb, Sandeep Arya, Viswanath Gajula, Mary B. Taylor, Bibhuti B. Das

**Affiliations:** 1 University of Mississippi Medical Center Program, Jackson, MS 39216, USA; hnannapaneni@umc.edu; 2 Department of Pediatrics, Division of Pediatric Cardiology, Children’s of Mississippi Heart Center, University of Mississippi Medical Center, Jackson, MS 39216, USA; sghaleb@umc.edu; 3 Department of Pediatrics, Division of Critical Care, Children’s of Mississippi Heart Center, University of Mississippi Medical Center, Jackson, MS 39216, USA; sarya@umc.edu (S.A.); vgajula@umc.edu (V.G.); mbtaylor@umc.edu (M.B.T.)

**Keywords:** neonatal dilated cardiomyopathy, familial dilated cardiomyopathy, RPL3L gene, gene mutation, neonatal heart failure

## Abstract

Neonatal dilated cardiomyopathy (DCM) is rare with high etiologic heterogeneity. Recently, biallelic, autosomal recessive, pathogenic variants in *RPL3L* (ribosomal protein L3-like) have been reported in the literature with severe early-onset DCM. In the present brief report, we identified two pathogenic *RPL3L* variants, each harbored in unaffected heterozygous parents: mother (*RPL3L c.1076_1080delCCGTG* (p.Ala359Glyfs*4)) and father (*RPL3L c.80G > A* (*p.Gly27Asp*)). Pathogenic variants were segregated as autosomal recessive to two offspring born with compound heterozygous RPL3L variants and affected by neonatal DCM. This is the second report in the literature to the best of our knowledge and our findings support the pathogenicity of biallelic *RPL3L* pathologic variants associated with rapidly progressive neonatal DCM and heart failure with a poor prognosis.

## 1. Introduction

Inherited and congenital heart diseases (CHDs) are leading causes of infant mortality. Traditionally, the diagnosis of cardiomyopathies and CHDs have relied on morphologic and functional findings on echocardiograms. Disease-specific gene panels using chromosomal microarray (CMA) technology are used as the first-line tests to address the genotypes among inherited cardiomyopathies [1]. If the gene panel test is negative, whole-exome sequencing (WES) or whole-genome sequencing (WGS) is considered a second-tier test [2,3]. More recently, PCR-free WGS has been found to offer proper coverage of the coding region of the genome as the most comprehensive second-tier test to diagnose inherited heart diseases [4]. Genome sequencing targets both the protein-coding and noncoding regions of the human genome, allowing for the possible detection of pathogenic variants in regions WES does not assess. The noncoding regions include promoter, intronic, and untranslated regions. While a large part of the data generated from genome sequencing is not well understood, WGS may provide more reliable coverage of the exonic regions [5,6]. However, WGS requires more sequencing reagents and produces extensive datasets that require sophisticated bioinformatics expertise to interpret, increasing the cost and time needed for analysis. On the other hand, WES is a cost-effective, widely used second-tier test that requires fewer sequencing reagents and takes less time to analyze than WGS. Although the human exome represents only 1–5% of the genome, it contains approximately 85% of known disease-related variants [7].

Several genes cause cardiomyopathy in a dominant inheritance pattern and rarely in recessive mode [8]. The diagnostic yield of conventional genetic panel testing for DCM is only 37%, whereas the next-generation sequencing (NGS) tests such as WES/WGS can increase the diagnostic yield up to 82.7% [9]. The reason for the high diagnostic product by NGS is that commercially available genetic panel tests would not test some of the newly reported gene variants. Al-Hassnan et al. have indicated that when there is a strong suspicion of a genetic etiology in a family with cardiac disease, NGS such as WES/WGS should be considered a first-line test [10]. We report autosomal recessively inherited compound heterozygous variants of *RPL3L* (ribosomal protein L3-like) in two affected siblings using WES. *RPL3L* variants were considered a variant of uncertain significance before 2020 because of a sporadic allele frequency distribution of <1 in 1000 in the general population and not predicted to be damaging, even though encoded proteins were highly expressed in heart muscles [10]. Our two cases represent only the second to previously reported five infants from three independent families presenting with severe familial neonatal DCM due to pathogenic RPL3L variants [11]. Furthermore, we summarized the genotypes and phenotypes of all familial neonatal DCM cases associated with RPL3L variants reported so far in the literature. As per our institutional review board protocol, we obtained consent from parents to publish de-identified data in this paper.

## 2. Case Presentation

**Figure 1:**
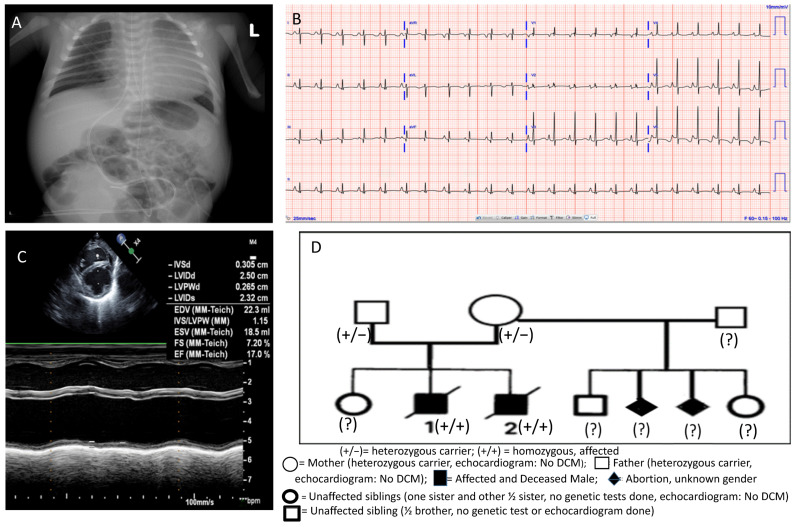
(**A**) Chest X-ray showing severe cardiomegaly on day 1 of life; (**B**) electrocardiogram showing nonspecific ST changes and biatrial enlargement; (**C**) M-mode echocardiography displaying left ventricular dilation and severely depressed function; (**D**) family pedigree showing RPL3L variant identification in family members.

A 30-year-old multiparous female presented at 36 2/7 weeks gestation with a pregnancy complicated by lack of prenatal care. She delivered a male neonate with a birth weight of 2.98 kg, length 52 cm, and head circumference of 33 cm. Physical examination and multisystem investigations excluded syndromic background. APGAR was 8 at one minute and 4 at five minutes since he was cyanotic with bradycardia (<80 bpm) and oxygen saturation in the mid-60s. Noninvasive respiratory support helped to increase oxygen saturation up to 90%. The neonate continued to be cold and clammy with intermittent grunting and was admitted to the neonatal intensive care unit (NICU). A chest X-ray showed severe cardiomegaly (Figure 1A). His EKG showed sinus rhythm with biatrial enlargement and nonspecific ST-segment abnormalities in the lateral leads (Figure 1B). Echocardiography (ECHO) showed left ventricular (LV) dilatation and biventricular depressed function (moderately depressed LV function with ejection fraction (LV EF) of 36%) (Figure 1C). Blood gas and other laboratory tests as surrogate markers of cardiac output were obtained as per standard of care, and the initial lactate level was elevated at 4.1.

Family history was significant for both parents born in South America, but not consanguineous. Figure 1D describes the family pedigree, including the proband, the other affected deceased sibling, two abortions, and three healthy siblings. Two years before the proband’s birth, a previous sibling also presented in cardiogenic shock at seven weeks of life. He was admitted to our pediatric intensive care unit (PICU), where an ECHO showed DCM and severely depressed LV function. He was placed on extracorporeal membrane oxygenation (ECMO) and died on the seventh day of admission due to multiorgan failure. The DCM genetic panel with CMA was unremarkable for that sibling at that time, and an autopsy was not done. Besides the above family history, the mother had two previous spontaneous abortions of unknown gender at two months of gestation from her prior marriage. The family history was also significant for a child with chronic heart failure in an extended family member on the paternal side (paternal aunt).

After the proband neonate was transferred to the PICU, he was evaluated for infectious (including Chagas disease given that both parents were from South America), metabolic, and autoimmune causes of DCM, and all results returned unremarkable. Inotropic support (milrinone) was started to improve cardiac output due to depressed LV function. His echocardiogram showed worsened biventricular function, and NT pro-BNP was elevated to 7000 pg/mL. At 5 days old, the milrinone infusion dose was further increased to 0.5 mcg/kg/min, epinephrine 0.01 mcg/kg/min was started, and the neonate was intubated for ventilator support.

We consulted the genetics team at our institution for the possibility of inherited cardiomyopathy due to the strong family history of cardiomyopathy. For the WES to be most effective, the buccal swabs from both parents and the proband (trio testing) were sent to GeneDx (Gaithersburg, MD, USA) for rapid XomeDxPlus. All biological samples were analyzed by Sanger sequencing at HudsonAlpha Institute for clinical services as per the SouthSeq research study (NIH research protocol 30000328) after obtaining appropriate consent from parents. The WES test resulted in the biallelic pathogenic variants in *RPL3L* that together are likely the reason for the proband’s DCM. The WES found *RPL3L c.1076_1080delCCGTG* (*p.Ala359Glyfsallele*) maternal allele and *RPL3L c.80G > A* (*p.Gly27Asp*) paternal allele. The male partner from the mother’s previous marriage and the half-brother of the proband were not living in the USA and thus unavailable for genetic and echocardiographic studies. The two sisters had echocardiograms as a part of screening for cardiomyopathy after the proband diagnosis and had normal cardiac examinations. Both carrier parents were unaffected and had normal echocardiograms. No CHD or arrhythmia in any unaffected siblings or parents was reported.

The patient was listed for heart transplant as status 1A in the United Network of Organ Sharing (UNOS) waiting list after the identification of the pathologic gene variants, previously reported as a cause of a severe form of neonatal DCM with an abysmal prognosis. His heart failure worsened while remaining intubated and on inotropic support. Parents refused ECMO and other forms of mechanical circulatory support and decided to withdraw ventilatory support. An autopsy was not performed per the parent’s request.

We tested whether the RPL3L variant was also present in the prior deceased sibling who had otherwise normal CMA and a negative genetic panel testing for DCM. The blood stored in the genetic testing laboratory was tested for *RPL3L* variants at HudsonAlpha Institute and included in the SouthSeq research protocol (protocol 30000328). The deceased affected sibling was also found positive for the same two pathological variants in the *RPL3L* gene, supporting a previous finding that the *RPL3L* gene is associated with familial neonatal DCM [11].

## 3. Meta-Analysis of All Seven DCM Cases Due to *RPL3L* Variants

The genotypes and phenotypes of all cases of DCM due to compound heterozygous variants of *RPL3L* mutations (including our current two instances) are summarized in Table 1. Details of the family history for Cases 1–5 are available in the original report by Ganapathi et al. [11]. One patient (Case 5) had an additional ventricular septal defect. Otherwise, no hemodynamically significant CHD, valvar diseases, or arrhythmia was reported in any of these cases who presented with severe DCM. Findings of a patent foramen ovale and pulmonary hypertension (Case 1) were normal findings for day 1 of life. Mitral regurgitation could be due to dilated left ventricle. Tricuspid regurgitation was reported in multiple cases and could be physiological unless associated with abnormal tricuspid valve morphology and right ventricle dilatation. The ST-T abnormalities reported in Cases 3–7 were usually signs of nonspecific changes due to DCM and myocardial dysfunction. Case 3 had right ventricular conduction delay could also be a nonspecific finding.

Two patients (Cases 3 and 4) underwent successful heart transplantations and were alive at age 9 and 10 years, respectively, at publication [11]. Early diagnosis of *RPL3L* variants can facilitate listing for a heart transplant. So far, those two reported cases had a somewhat normal post-transplant course. Case 3 developed lymphoproliferative disease following heart transplantation. There was no information available on the use of mechanical circulatory support to manage advanced decompensated heart failure. Our proband (Case 6) was listed for a heart transplant and died while on the waiting list. Parents refused to use mechanical circulatory support. Case 7 was on ECMO support and died due to multiorgan failure. There was no family history of consanguinity except in Case 3. In all seven patients in four families, including ours, parents and unaffected siblings had no cardiomyopathy or arrhythmia reported. 

## 4. Discussion

WES in parent-offspring trios provides an efficient means to discover the genetic basis of DCM and ultimately enhances the yield of molecular diagnostics, as demonstrated in our case, which supports the previous findings reported by prior studies [9,10,11]. Pathogenic variants of the *RPL3L* sequence associated with neonatal DCM in both siblings in our report were determined by the criteria laid out by the American College of Medical Genetics and Genomics and the Association for Molecular Pathology [12]. Similar to our two cases, previously, Ganapathi et al. [11] identified causative, compound heterozygous missense variants in RPL3L in severe neonatal form of DCM patients using trio-based WES. The identified variants cosegregated with the disease in each of the three families and were absent or very rare in the human population, in line with an autosomal recessive inheritance pattern. The detailed pathomechanisms underlying *RPL3L*-associated DCM are not known. The *RPL3L* mutation is fascinating since it is the first ribosomal gene implicated in cases of DCM. Ribosomes containing *RPL3L* have altered translational activity, and it has been postulated that *RPL3L* may be a negative regulator of muscle growth [13]. The *RPL3L* gene codes for the skeletal and heart muscle-specific 60S ribosomal subunits. Genomic sequencing has shown the *RPL3L* variants can cause destabilization of the 60S subunits and interfere with ribosomal translation, leading to neonatal DCM [11]. *RPL3L* sequence variants with the missense variant *p.Ala75Val* and the splice-donor variant *c.1167 + 1G > A* have also been implicated in atrial fibrillation [14]. The speculated mechanism of atrial fibrillation is that *RPL3L* gene mutation may prolong the P-wave duration, alter atrial conduction, and cause possible atrial fibrillation [15]. Although these findings suggest the *RPL3L* can affect the cardiac myocyte function and also electrical activity of the heart, no arrhythmias have been reported in DCM cases so far reported in the literature.

The ability to promptly diagnose a neonate with a genetically caused cardiomyopathy helps the clinicians discuss with the family various options, including palliative care, especially when a genetic diagnosis has a known fatal outcome. Furthermore, a genetic diagnosis can prevent the need for other invasive procedures such as skeletal muscle or skin biopsy, cardiac catheterization, and endomyocardial biopsies and thus can be cost-effective. Our experience with only one family included in this report suggests WES can improve the molecular diagnostic yield as reported previously compared to the conventional genetic panel tests for DCM. Early identification of a pathologic variant gene association with DCM is helpful for risk stratification, management, and informed family counseling.

## 5. Conclusions

We have presented autosomal recessive inheritance of RPL3L pathologic variants *RPL3L c.1076_1080delCCGTG* (p.Ala359Glyfs*4) from the mother and *RPL3L c.80G > A* (*p.Gly27Asp*) from the father resulting in compound heterozygous segregation in two offspring and rapidly progressive fatal neonatal DCM. WES is helpful to identify the etiology of DCM where etiology is unknown, especially in a nonsyndromic DCM patient, and is essential for management. We suggest that these pathologic variants might be considered in gene panels for cardiomyopathy studies in those centers that cannot afford exome sequencing. Further studies are needed to analyze the detailed pathomechanisms underlying *RPL3L*-associated DCM.

## Figures and Tables

**Table 1:** Clinical and genetic findings in seven patients (including two present cases) with compound heterozygous variants of *RPL3L* diagnosed using WES and associated with the neonatal form of severe DCM reported in the literature.

Case No.	Sex	Consanguinity	Age at Diagn. of DCM	Country	Additional Cardiac Findings	Alive or Dead	Variants Identified	Reference
1	F	No	Day 1	Germany	PFO PH	Died 21st DOL	*c.923A > T* (*p.Asp308Val*) and *c.1027C > T* (*p.Arg343Trp*)	[11]
2	M	No	Day 6	Germany	TR	Died 15th DOL	*c.923A > T* (*p.Asp308Val*) and *c.1027C > T* (*p.Arg343Trp*)	[11]
3	M	Yes	Day 75	Colombia	TR, MR RBBB ST-T abnormalities	HT at 6 months Alive at 9 years	*c.566C > T* (*p.Thr189Met*) and *c.922G > A* (*p.Asp308Asn*)	[11]
4	F	No	Day 48	Spain	MR ST and T abnormalities	HT at 5 months Alive at 10 years	*c.80G > A* (*p.Gly27Asp*) and *c.481C > T* (*p.Arg161Trp*)	[11]
5	M	No	Day 12	Spain	MR, TR ST and T abnormalities VSD	Died 30th DOL	*c.80G > A* (*p.Gly27Asp*) and *c.481C > T* (*p.Arg161Trp*)	[11]
6	M	No	Day 1	USA	MR ST abnormalities	Died 124th DOL	*c.1076_1080delCCGTG* (p.Ala359Glyfs*4) and *c.80G > A* (*p.Gly27Asp*)	Present
7	M	No	Day 49	USA	MR ST abnormalities	Died 56th DOL	*c.1076_1080delCCGTG* (p.Ala359Glyfs*4) and *c.80G > A* (*p.Gly27Asp*)	Present

No. = number; M = Male; F = Female; DCM = dilated cardiomyopathy, Diagn. = diagnosis; PFO = patent foramen ovale, PH = pulmonary hypertension; TR = tricuspid regurgitation; MR = mitral regurgitation; ST: a segment in ECG from the beginning of S wave until the beginning of T wave; RBBB = right bundle branch block; DOL = day of life; HT = heart transplant.

## Data Availability

Not applicable.
